# Impact of SA-related visual characteristics on driver takeover reaction time in L3 automated vehicles: an eye-movement-based evaluation method

**DOI:** 10.3389/fpsyg.2026.1881353

**Published:** 2026-08-26

**Authors:** Jian Wan, Yue Zeng, Huayu Liu, Yulin Ma

**Affiliations:** 1 School of Software Engineering, Jinling Institute of Technology, Nanjing, Jiangsu, China; 2 Jiangsu Key Laboratory of Intelligent and Low-Carbon Transportation, Nanjing, Jiangsu, China; 3 School of Mechanical and Automotive Engineering, Anhui Polytechnic University, Wuhu, Anhui, China

**Keywords:** automated vehicle, driving simulator, eye-tracking, machine learning, situation awareness, takeover performance

## Abstract

**Introduction:**

In Level 3 (L3) conditionally automated driving, drivers must rapidly regain situational awareness (SA) following a takeover request. This study examined pre-takeover eye-movement characteristics and their associations with SA-related states and takeover reaction time under different takeover scenarios and non-driving-related tasks (NDRTs).

**Methods:**

A driving simulator experiment was conducted under four conditions. Of 168 trials from 42 participants, 108 valid trials from 27 participants were analyzed. Pupil area, saccade duration, and fixation duration during the 5 s before takeover were integrated into an SA-related score using the CRITIC method. Machine-learning models were compared to predict takeover reaction time.

**Results:**

NDRT type significantly affected the three eye-movement indicators, whereas takeover scenario did not significantly affect these indicators or reaction time. The SA-related score was significantly associated with takeover reaction time. LightGBM achieved the best predictive performance, with an accuracy of 0.818 and an F1-score of 0.837, and the SA-related score showed the highest predictive contribution (29.40%).

**Discussion:**

Pre-takeover eye-movement characteristics may provide an objective physiological proxy for SA-related visual-processing states and support the prediction of initial takeover response speed. These findings may inform adaptive driver monitoring and takeover assistance, although the observed relationships are predictive rather than causal.

## 1 Introduction

Human error remains the primary catalyst for traffic accidents, accounting for over 90% of crashes (Shinar, 2017). The rapid advancement of automated driving technologies offers a promising solution to mitigate human-induced risks and enhance both road safety (Parekh et al., 2022) and traffic efficiency (Taiebat et al., 2018). However, under Level 3 (L3) conditional automation, the driver’s role fundamentally shifts from an active operator to a passive supervisor. This transition significantly increases the likelihood of drivers falling “out-of-the-loop” (Merat et al., 2019) and engaging in non-driving-related tasks (NDRTs). Upon receiving a takeover request (TOR), drivers must rapidly reconstruct their situational awareness (SA) to make critical driving decisions (Yi-Jing et al., 2019). Unfortunately, NDRTs degrade the driver’s baseline SA of the dynamic road environment (Shi and Frey, 2021). The time lag required for SA reconstruction directly translates to increased safety risks, making the evaluation of SA a pivotal metric in assessing takeover readiness and reaction time.

Endsley (Endsley, 1988) defined SA as the perception of environmental elements, comprehension of their meaning, and projection of future states. While direct measurement techniques, such as the situation awareness global assessment technique (SAGAT) (Endsley, 1995) and the situation present assessment method (SPAM) (Durso et al., 1998), have been widely used, they can be intrusive. Consequently, indirect physiological measures have gained traction for being more objective and continuous (Nguyen et al., 2019). Specifically, eye-movement characteristics—such as blink rate, gaze direction, and fixation duration—have proven highly effective in real-time driver state monitoring (Jin et al., 2013; Zhang et al., 2023). Recent studies have further validated the robust link between eye movements and SA; for instance, Zhou et al. (2021) and Lu et al. (2017) successfully utilized eye-tracking data to model SA levels, establishing eye-movement metrics as a reliable foundation for SA quantification.

The determinants of SA and takeover time are multifaceted, generally categorized into driver-centric attributes and environmental constraints. Existing literature indicates that SA is heavily influenced by cognitive load, age, and driving experience (Lu et al., 2021; Vlakveld et al., 2018; Yang et al., 2020), while takeover time is modulated by TOR lead times, request modalities, and specific traffic scenarios (Dogan et al., 2019; Roche et al., 2019; Vlakveld et al., 2018). During the transition of control, reconstructing the mental representation of a dynamically changing traffic situation incurs a cognitive cost. Extensive research confirms that higher cognitive costs during SA recovery inevitably lead to prolonged reaction times and degraded takeover quality (Marti et al., 2022; Naujoks et al., 2018).

Despite these advancements, critical gaps remain in the current understanding of SA during the takeover process. First, previous studies often treat eye-movement characteristics as isolated independent variables, neglecting the underlying interaction mechanisms among different visual indicators. Second, while physiological indicators have been correlated with SA, a standardized, quantitative SA evaluation framework based on these interactions is still lacking. Finally, there is a scarcity of research that accurately quantifies the individual contribution of these influencing factors to takeover time.

To address these gaps, this study conducts a driving simulator experiment under L3 automation to investigate pre-takeover eye-movement characteristics and their impact on takeover reaction time. The primary contributions of this research are fourfold: (1) Analyzing the differential impacts of various driving scenarios and NDRTs on visual behaviors and takeover time; (2) Elucidating the interconnections and interaction mechanisms among key eye-movement indicators; (3) Developing an eye-movement-based SA-related evaluation method using weighted physiological features; and (4) Constructing a LightGBM-based predictive model to identify and quantify the key factors governing reaction time. Ultimately, this research provides a theoretical foundation for the real-time monitoring of driver SA, risk assessment during transitions of control, and the design of safer human-machine interfaces.

## 2 Methodology

### 2.1 Driving simulation experiment

#### 2.1.1 Experiment platform

A comprehensive test platform for automated driving takeover behavior was developed using the high-fidelity driving simulation system at Beijing University of Technology. The platform leverages SCANeR™ studio 1.9 software for road network construction, dynamic scenario simulation, and the implementation of L3 automated driving functionalities.

**FIGURE 1:**
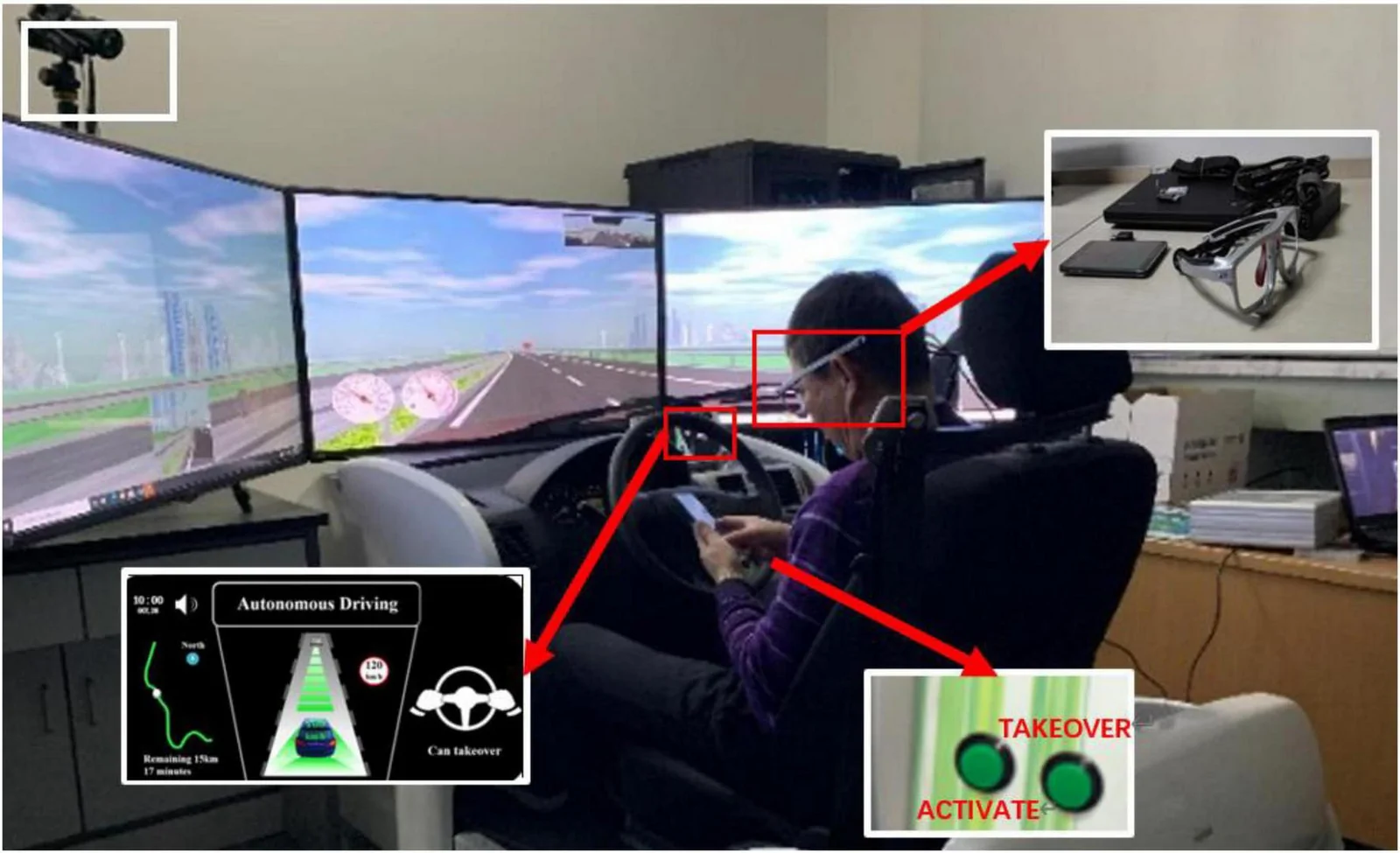
Automatic driving takeover behavior test platform.

During the simulation, driving state information and takeover guidance are presented to the driver via an on-board HMI. Real-time data transmission between the central data cooperative processing unit and the intelligent on-board terminal is achieved through a high-speed wireless communication network. To capture drivers’ visual attention and cognitive load, aSee Glasses—a wearable eye-tracker with a 60 Hz sampling rate—were utilized to record precise eye-movement data and high-resolution scene videos. The seamless integration and synchronization of these hardware and software components constitute the automated driving takeover test platform (Figure 1).

#### 2.1.2 Experiment design

Takeover-request lead time (TORlt) refers to the time interval between the issuance of a takeover request and the predefined critical event location. Previous studies have shown that a TORlt of 5 s can provide drivers with sufficient time to initiate a takeover response without inducing excessive reliance on the automated driving system (Zeeb et al., 2015). Therefore, the TORlt was set to 5 s in this experiment based on previous findings, system constraints, and driving safety considerations. It should be noted that TORlt is distinct from takeover reaction time, which was defined in this study as the interval between the warning trigger and the driver pressing the “takeover” button.

The no-driving-related task (NDRT) refers to the tasks unrelated to driving activities performed by the driver during autonomous driving. The work task state and the recreational task state are two types of task states that are often used (Dogan et al., 2019), forming different degrees of workload on the driver’s visual and auditory cognition. In this experiment, we selected the entertainment task as watching entertainment videos and answering questions, and the work task as reading news and sending WeChat messages, taking into account the actual situation of drivers.

**FIGURE 2:**
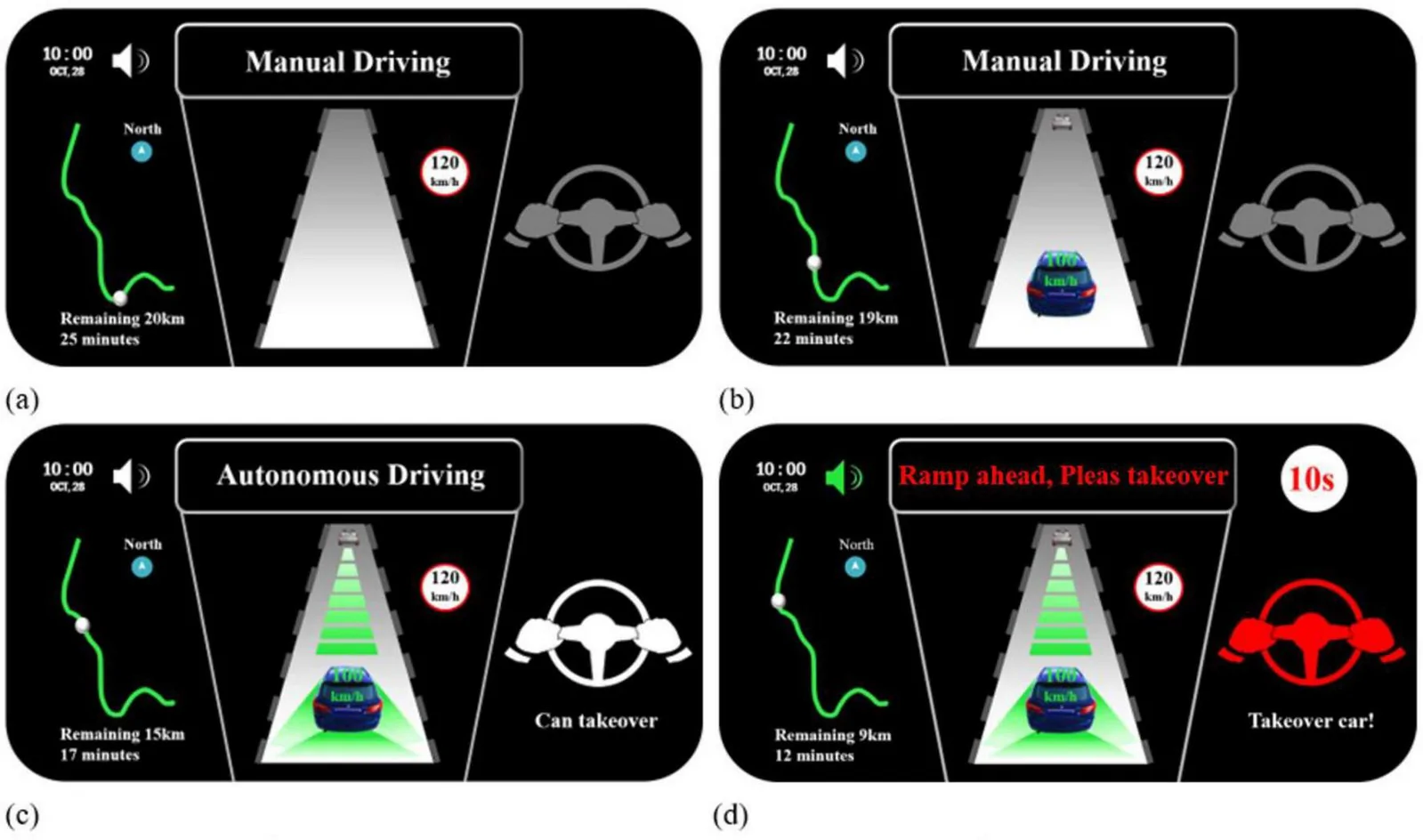
HMI interface design (Ramp takeover as an example). **(a)** Manual driving-AVs is not available. **(b)** Manual driving-AVs is available. **(c)** Automated driving-AVs is activated. **(d)** Automated driving-AVs need to be takeover.

In the process of takeover, the human-machine interface (HMI), which realizes the control conversion between the automatic vehicle and the driver, is one of the crucial factors affecting the takeover behavior. In this study, the intelligent terminal uses a fixed touch-screen tablet computer to build a data interaction platform that can meet the requirements of sending takeover warning information. Based on the previous HMI design standards, design experience and user experience feedback, the HMI interface is set to four states according to the automatic driving function state and driving mode. HMI provides vehicle operation, takeover status information and audio-visual takeover warning, providing drivers with relevant information and tips while minimizing the driver’s distraction level (Figure 2). For example, 5s before the automatic driving system triggers the ramp takeover event, it sends drivers a takeover request message of “Ramp ahead, please takeover” with an emergency prompt tone. Drivers need to press the “takeover” button to takeover control of the vehicle, and press the “activate” button to start the automatic driving when the automatic function is restored (Figure 1).

**FIGURE 3:**
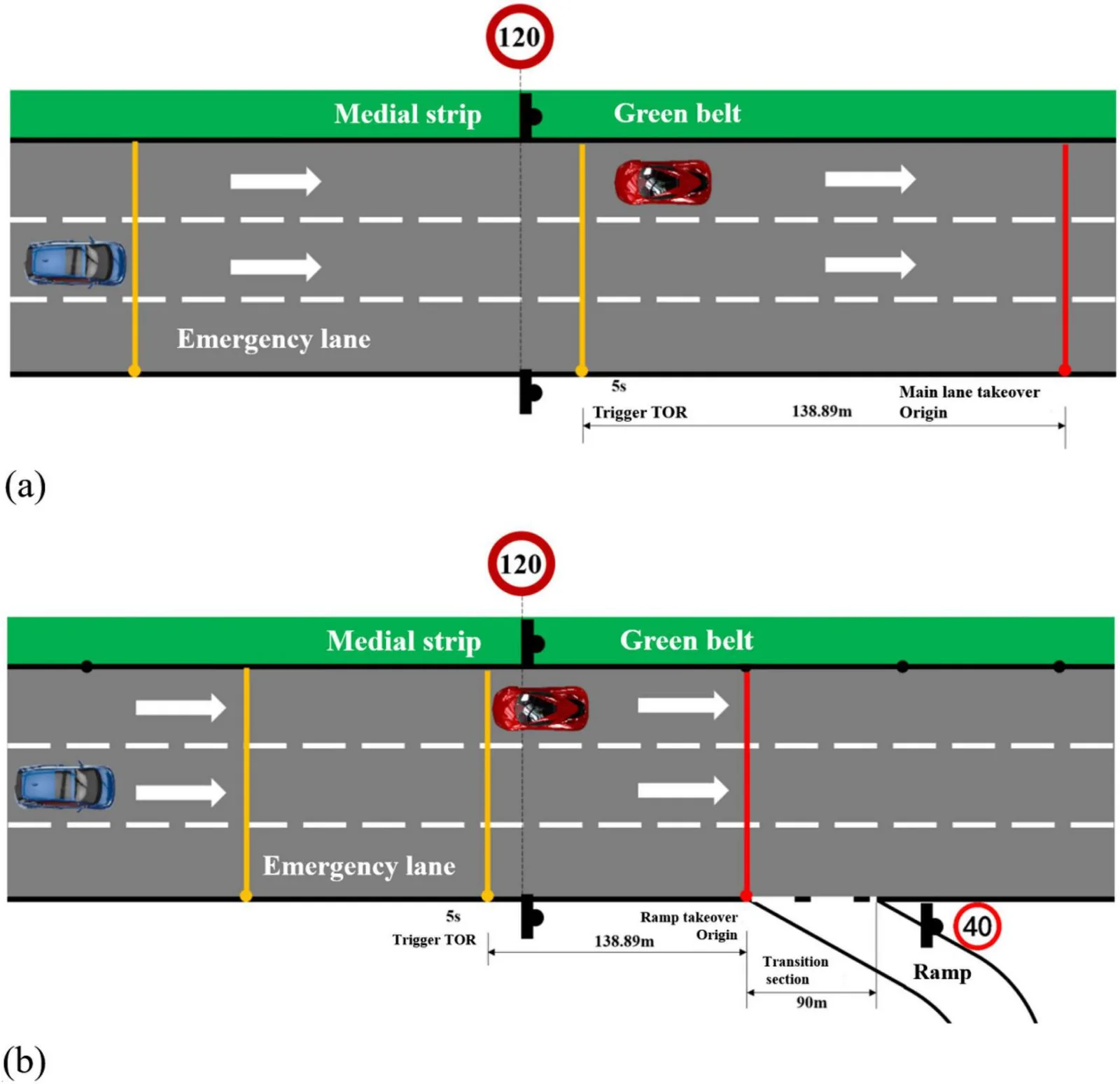
Spatial design of takeover scenes. **(a)** Main lane takeover spatial position parameter. **(b)** Ramp takeover spatial position parameter.

Road simulation scenarios are developed by 3ds Max and HintCAD. The road type is a two-way four-lane highway (lane width 3.75 m, shoulder 4.5 m), the road speed limit is 120 km/h in the free flow state, and the speed is set to 100 km/h by speed downgrade during automatic driving. Drivers decide the speed after taking over the vehicle according to personal experience. In this experiment, under the TOR condition of 5 s, the early warning trigger position is 138.89 m before the preset starting point of the event (Figure 3). Takeover events include: (1) Main line takeover: as the control group of the experiment design, there is no event ahead. Driver needs to take over the control of the vehicle when the vehicle announces the warning “Please takeover” according to the preset warning time. (2) Ramp takeover: there is a ramp exit on the right side of the road ahead to enter the service area. Driver needs to take over and enter the ramp when the vehicle warns “Ramp ahead, please takeover” according to the preset warning time.

This study designed four takeover scenario programs based on the combination of two factors: takeover scenario (main line takeover S1, ramp takeover S2) and NDRT (entertainment task N1, work task N2). Considering the correlation between takeover scenarios, four takeover scenes are distributed on two road segments with obvious differences design styles according to NDRT. There is a long enough distance between different takeover scenarios to reduce the learning effect.

#### 2.1.3 Participants and experimental procedure

Forty-two subjects were recruited from universities and social groups, of whom 76.2% were male and 23.8% were female. The average age of the participants was 42.53 years, and the average driving experience was 17.06 years (Table 1). All the subjects meet the test criteria of the driving simulator experiment.

**TABLE 1:** Driver information statistics.

Driver attributes	Attribute level		Average	Standard deviation
Gender	Male(32)		–	–
	Female(10)			
Age	Young (15)	18–35	42.53	15.85
	Middle-aged(14)	36–60		
	Elderly(13)	>60		
Driving experience	Low(18)	1–12	17.06	12.17
	Medium(14)	13–26		
	High(10)	27–42		

The number of people is in parentheses and the age range is on the right.

The experimental procedure is as follows. Participants filled out a basic questionnaire before the experiment began. Then the training of the experimental process, the operation of the driving simulator and the operation of the automatic driving takeover is carried out. In the first experiment, a road section was randomly selected to start the driving simulation experiment, and the data was collected. Then the driver has a rest period of 10–15 min. In the second experiment, chose another road section and repeated the experiment. At the end of the test, the post-experiment questionnaire was filled in and the experiment reward was paid. All participants provided written informed consent before the experiment. The study adhered to anonymity and data protection principles, safeguarding participants’ rights to information, privacy, and voluntary withdrawal.

### 2.2 Indicators

The experiment evaluated drivers’ eye-movement behavior during the pre-takeover phase and takeover reaction time after the takeover request. Raw eye-movement signals were recorded using an eye tracker with a sampling frequency of 60 Hz. To synchronize the eye-movement data with other experimental signals and reduce high-frequency noise, the raw signals were preprocessed and resampled to 20 Hz for subsequent indicator extraction. The extracted indicators are described as follows:

(1) Pupil area (*Sp*): the average pupil area of the driver within 5s before the takeover request is issued, which is used to represent the visual workload of the driver.
(2) Saccade duration (*Ts*): the average of the driver’s scanning duration within 5 s before the takeover request is issued, which characterizes driver’s ability to find key information.
(3) Fixation duration (*Tf*): the average of the driver’s gaze duration within 5s before the takeover request is issued, which represents the driver’s ability to understand and extract key information.
(4) Reaction time (*Tr*: refers to the time between the automatic driving vehicle passing the trigger point of each event warning and the driver pressing “takeover” button, representing the driver’s takeover response speed.

### 2.3 Data analysis method

A total of 168 experimental trials were collected from 42 participants under four driving simulation conditions. During data preprocessing, invalid trials were excluded according to predefined data quality criteria, including incomplete eye-tracking recordings, signal loss, and abnormal experimental responses. In addition, observations exceeding three standard deviations from the participant-level distribution were examined as potential outliers. After data quality control, 108 valid trials from 27 participants were retained for subsequent analysis. The exclusion procedure was based on data validity rather than takeover performance or experimental conditions to minimize potential selection bias.

When analyzing the experimental data, the Shapiro-Wilk test was used to test the normality of the data. Since the experimental data followed a within-subject repeated-measures design, the Friedman test was employed to examine differences among experimental conditions for non-normally distributed variables. When significant differences were detected, post-hoc pairwise comparisons with appropriate correction were conducted to identify differences between conditions.


$$W=\frac{\chi_{F}^{2}}{n\,\left(k-1\right)}$$ (1)

The effect size of the Friedman test was evaluated using Kendall’s coefficient of concordance (W), as calculated using Equation 1, which reflects the magnitude of differences among repeated measurements. The value of W ranges from 0 to 1, with larger values indicating stronger effects. According to commonly used criteria, W values of 0.1, 0.3, and 0.5 indicate small, moderate, and large effects, respectively.

K-Means clustering was used for all clustering methods of the data, and contour coefficients were used to evaluate the superiority of the clusters. Kendall’s W test was used in analyzing the correlation of the eye movement indicators to obtain the concordance coefficient taking values between 0 and 1, which is used to measure the consistency between different measurements (Li et al., 2008). For the calculation of the weights of the metrics, the CRITIC weighting method was used, which is an objective weighting approach that determines indicator importance by considering both the contrast intensity and conflict among evaluation criteria (Diakoulaki et al., 1995). Compared with subjective weighting approaches, such as the analytic hierarchy process (AHP), CRITIC avoids the influence of expert preferences and provides data-driven weights. Moreover, unlike entropy-based weighting methods that mainly focus on information uncertainty, CRITIC additionally considers the correlations among indicators, making it suitable for integrating multiple eye-movement features with potential interactions in SA evaluation.

In the reaction time prediction process, a variety of machine learning classification models were compared, such as Random Forest and Extra Trees algorithms based on constructing decision trees, GBDT based on gradient boosting, CatBoost that improves accuracy and generalization on GBDT, XGBoost that optimizes the loss function and improves the efficiency, and LightGBM to further improve the efficiency of XGBoost.

To evaluate the predictive capability and generalizability of machine learning models, the dataset was randomly divided into training and testing subsets with a ratio of 80 and 20%, respectively. The splitting process was performed using a fixed random seed to ensure reproducibility. The training subset was used for model development, while the testing subset was exclusively used for final performance evaluation. Considering the relatively limited sample size obtained from the driving simulator experiment, a five-fold cross-validation strategy was further applied within the training dataset to reduce the influence of random partitioning and improve model robustness. The average performance across five validation folds was used for model selection and parameter optimization. Finally, the optimized models were evaluated using the independent testing dataset based on accuracy, recall, precision, and F1-score.

## 3 Results

This chapter presents the results of the experimental analysis. First, the variations in eye-movement indicators and takeover reaction times under different takeover scenarios and NDRTs are examined using appropriate statistical tests. Second, the associations among eye-movement indicators are investigated through clustering and Kendall’s W analysis. Third, an eye-movement-based SA-related score is constructed using the CRITIC weighting method, and its variations across different experimental conditions are analyzed. Finally, the performance of different machine learning classification models is compared, and the LightGBM model is applied to evaluate the relative contribution of different factors associated with takeover reaction time.

### 3.1 Statistical analysis of eye-movement indicators and reaction time under different experimental conditions

A total of 168 experimental trials were collected from 42 participants under four driving simulation conditions. After excluding invalid trials and abnormal observations identified through boxplot inspection, 108 valid observations from 27 participants were retained for subsequent analysis. The Shapiro–Wilk test indicated that the eye-movement indicators and takeover reaction time did not satisfy the normality assumption. Considering that each participant completed multiple experimental conditions, the dataset followed a within-subject repeated-measures design. Therefore, the Friedman test was employed to examine the statistical differences among experimental conditions, and Kendall’s coefficient of concordance (W) was adopted to evaluate the effect size.

The statistical results are summarized in Table 2. No significant differences were observed among different takeover scenarios for pupil area, saccade duration, fixation duration, or reaction time (all *p* > 0.05). These results indicate that the takeover scenario did not significantly influence drivers’ visual processing characteristics or takeover reaction time. In contrast, different NDRTs significantly affected several eye-movement indicators, including pupil area, saccade duration, and fixation duration, while no significant difference was observed in takeover reaction time.

**TABLE 2:** Significance test and effect size results of eye-movement indicators and reaction time.

Indicator	Shapiro–Wilk test	Takeover scenario	NDRT
	W, p	χ^2^, p, W	χ^2^, p, W
*Sp*	0.923, < 0.001***	χ^2^ = 0.259, *p* = 0.611, *W* = 0.007	χ^2^ = 6.480, *p* = 0.011**, *W* = 0.034
*Ts*	0.622, < 0.001***	χ^2^ = 2.099, *p* = 0.147, *W* = 0.010	χ^2^ = 14.480, *p* < 0.001***, *W* = 0.022
*Tf*	0.862, < 0.001***	χ^2^ = 2.529, *p* = 0.112, *W* = 0.022	χ^2^ = 4.083, *p* = 0.043**, *W* = 0.023
*Tr*	0.959, 0.001***	χ^2^ = 1.708, *p* = 0.191, *W* = 0.014	χ^2^ = 0.194, *p* = 0.659, *W* = 0.007

χ^2^ represents the Friedman test statistic. W represents Kendall’s coefficient of concordance as the effect size.

**p < 0.05, ***p < 0.01.

**FIGURE 4:**
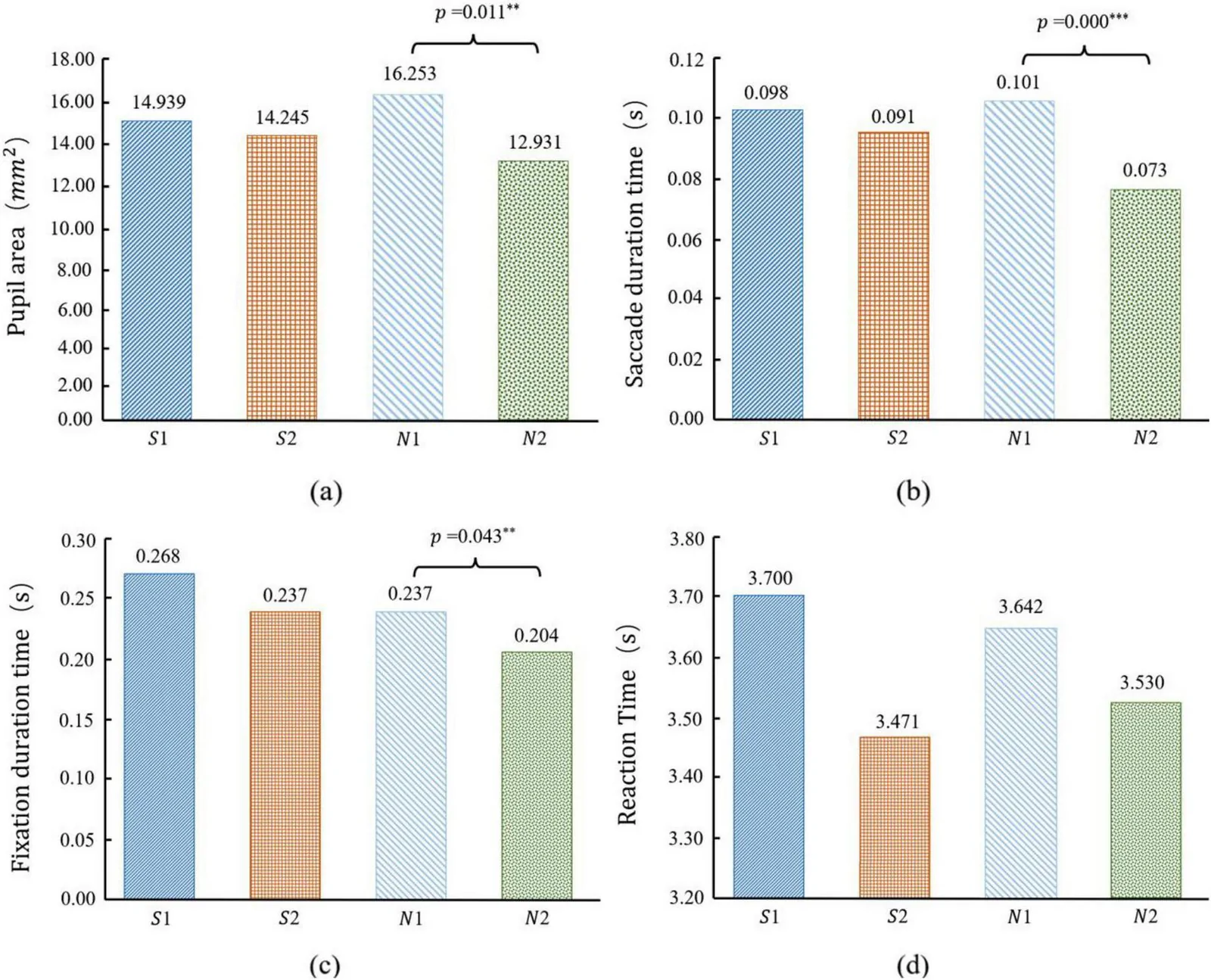
Statistical analysis of eye movement indicators and reaction time in different situations. **(a)** Pupil area. **(b)** Saccade duration time. **(c)** Fixation duration time. **(d)** Reaction time.

Figure 4 illustrates the effects of different experimental conditions on drivers’ eye-movement characteristics and takeover reaction time. Regarding takeover scenarios, no significant differences were found in pupil area (χ^2^ = 0.259, *p* = 0.611, *W* = 0.007), saccade duration (χ^2^ = 2.099, *p* = 0.147, *W* = 0.010), fixation duration (χ^2^ = 2.529, *p* = 0.112, *W* = 0.022), or reaction time (χ^2^ = 1.708, *p* = 0.191, *W* = 0.014). This suggests that variations in takeover location did not significantly alter drivers’ visual information processing or takeover response behavior.

In comparison, NDRT significantly influenced drivers’ eye-movement patterns. Specifically, significant differences were observed in pupil area (χ^2^ = 6.480, *p* = 0.011, *W* = 0.034), saccade duration (χ^2^ = 14.480, *p* < 0.001, *W* = 0.022), and fixation duration (χ^2^ = 4.083, *p* = 0.043, *W* = 0.023). As shown in Figures 4a–c, drivers performing different NDRTs exhibited different visual information acquisition and processing behaviors before takeover. However, no significant difference was detected in reaction time (χ^2^ = 0.194, *p* = 0.659, *W* = 0.007), indicating that the influence of NDRT was mainly reflected in pre-takeover visual processing rather than the final takeover response time.

Overall, the results indicate that NDRTs induced significant variations in drivers’ visual workload and information-processing characteristics during the pre-takeover phase. In contrast, takeover scenarios did not significantly affect either eye-movement indicators or reaction time. These findings suggest that the cognitive demand associated with secondary tasks played a more important role than takeover location in influencing drivers’ visual behaviors before takeover.

### 3.2 Relationships within eye movement indicators

Pupil area reflects drivers’ visual workload, fixation duration represents information processing and comprehension characteristics, and saccade duration reflects visual information searching behavior. No obvious linear relationships were identified among the original continuous eye-movement indicators, which may be attributed to the fact that these measures represent different aspects of visual information processing. To further investigate the potential associations among these indicators, the three eye-movement variables were first classified using K-means clustering. Each indicator was divided into three categories, namely low, medium, and high levels, as shown in Table 3.

**TABLE 3:** Eye movement indicator clustering results.

Indicators	Clustering category			*p*
	Low	Medium	High	
*Sp*	[7.98, 16.3]	[16.3, 29.7]	[29.7, 35.0]	< 0.001
*Ts*	[0.143, 0.221]	[0.221, 0.338]	[0.338, 0.430]	< 0.001
*Tf*	[0.066, 0.086]	[0.086, 0.131]	[0.131, 0.183]	< 0.001

The continuous values of the three indicators were converted into categorical labels to conduct pairwise association analysis. Kendall’s W analysis was subsequently performed to examine the consistency among different eye-movement indicators. The results showed that pupil area and fixation duration exhibited a significant but weak association (*W* = 0.040, *p* < 0.05). Similarly, saccade duration and fixation duration showed a significant but weak association (*W* = 0.056, *p* < 0.05), whereas no significant association was observed between pupil area and saccade duration.

### 3.3 Method of constructing SA evaluation

The three eye-movement indicators considered in this study were extracted during the pre-takeover phase and were expected to provide physiological information reflecting drivers’ SA-related visual processing characteristics during takeover preparation. Since these indicators characterize different aspects of visual information processing and may contain correlated information, an objective weighting strategy was required to integrate them into a comprehensive SA-related score. It should be noted that the proposed SA-related score represents an eye-movement-based estimation of drivers’ SA-related state rather than a direct measurement of SA as defined by Endsley. Therefore, since the eye-movement indicators represent different aspects of visual information processing and may contain partially overlapping information, an objective weighting method was required to determine their relative contributions. Therefore, the CRITIC weighting method proposed by Diakoulaki et al. (1995) was adopted to determine the relative importance of each indicator. CRITIC is an objective weighting approach that considers both indicator variability and inter-indicator conflict, thereby reducing subjective bias and preserving the inherent information contained in multiple physiological features.

Based on the obtained weights, pupil area, saccade duration, and fixation duration were integrated using a weighted aggregation approach. The resulting SA score was subsequently normalized to a range of 0–1, where 0 and 1 represent the lowest and highest SA levels, respectively.

**FIGURE 5:**
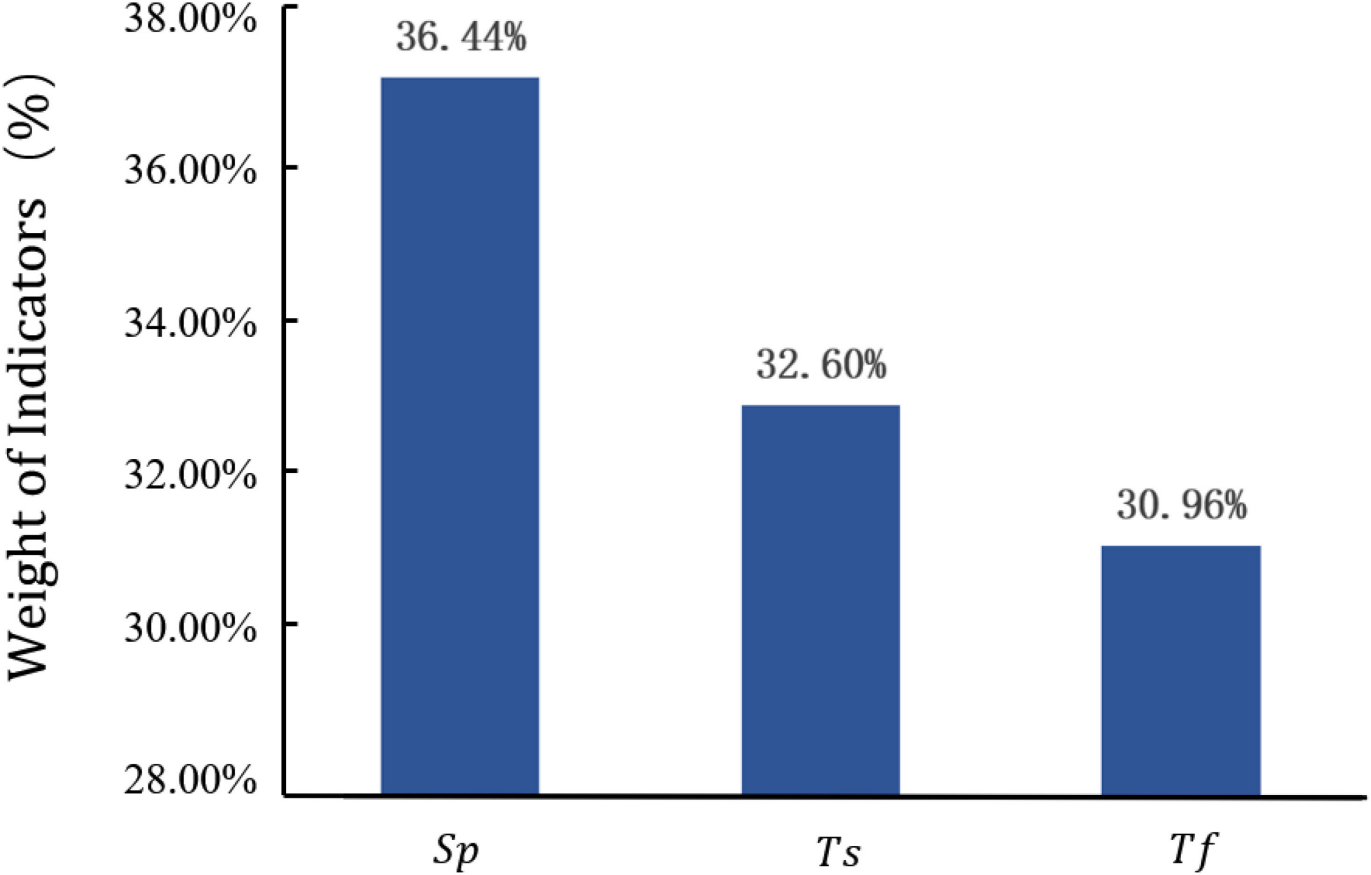
Weights of the eye-movement indicators used to construct the SA-related score.

The results showed that pupil area was weighted at 36.44%, saccade duration at 32.60%, and fixation duration at 30.96% (Table 4 and Figure 5). Considering the different relationships between eye-movement indicators and SA-related states, pupil area and fixation duration were regarded as negative indicators, whereas saccade duration was regarded as a positive indicator. Specifically, a larger pupil area indicates greater visual workload, which is associated with a lower SA-related state. A longer saccade duration reflects stronger information-searching ability and is associated with a higher SA-related state. In contrast, longer fixation duration indicates greater difficulty in information processing and is associated with a lower SA-related state. Therefore, considering the positive and negative characteristics of these indicators, the directions of the indicators were adjusted before applying the weighted average method to obtain the SA-related score (Equation 2). The formula for normalization is given as follows (Equation 3).


$$S\,A_{i}=\frac{-S\,p_{i}\times 36.44\%+T\,s_{i}\times 32.6\%-T\,f_{i}\times 30.96\%}{3}$$ (2)

$${\bar{S\,A}}_{i}=\frac{x_{i}-\text{min}\,\left(x_{i}\right)}{\text{max}\,\left(x_{i}\right)-\text{min}\,\left(x_{i}\right)}$$ (3)

**TABLE 4:** Calculation of weights for indicators.

Indicators	S	R	C	W(%)
*Sp*	0.212	2.075	0.439	36.440
*Ts*	0.192	2.042	0.393	32.604
*Tf*	0.197	1.892	0.373	30.955

S is the index variability, R is the index conflict, C is the information content, and W is the weight.

Where *SA*_*i*_ denotes the initial SA-related score and ${\bar{S\,A}}_{i}$ denotes the normalized SA-related score. To better characterize the variation in SA-related states, the normalized scores were clustered into low, medium, and high SA-related score groups, containing 37, 52, and 19 datasets, respectively.

**FIGURE 6:**
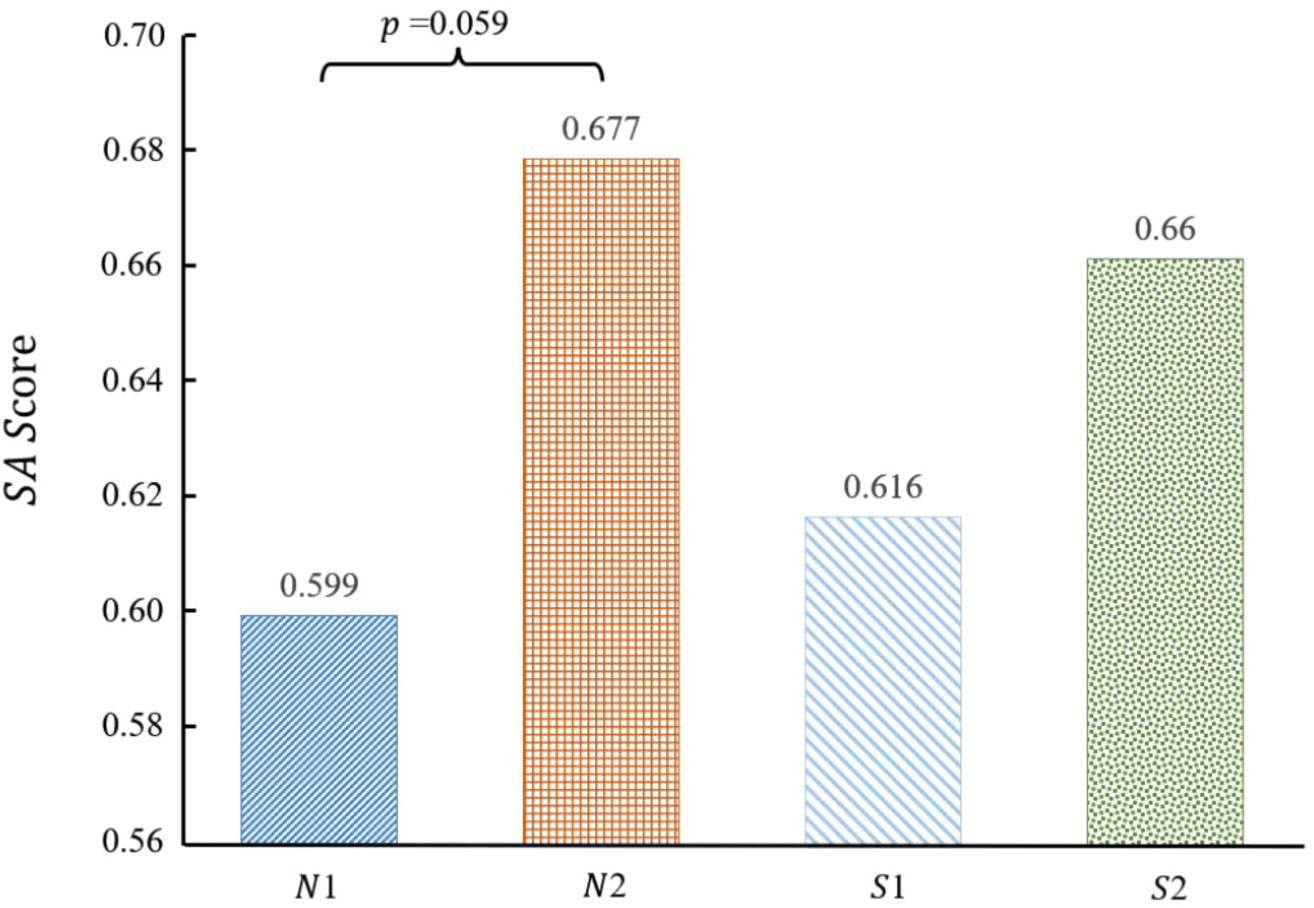
Statistical analysis of SA-related scores under different programs.

The statistical analysis showed no significant difference in SA-related scores between the two NDRT conditions (p = 0.059). No significant difference was observed between the two takeover scenarios (Figure 6). Therefore, the variation in SA-related scores across NDRT conditions should be interpreted cautiously. Spearman’s correlation analysis indicated a statistically significant association between the SA-related score and takeover reaction time (*p* < 0.05). This result suggests that pre-takeover visual-processing characteristics represented by the SA-related score were associated with takeover response speed.

### 3.4 Exploring the key factors affecting takeover reaction time

Classification model analysis of machine learning can help to explore the key factors affecting reaction time. In the factor extraction, this study considered three aspects: (1) driver SA situation, (2) basic driver attributes, including gender, age and driving age, (3) takeover environment, including different takeover scenarios and NDRT. Different machine learning models are compared in the process of constructing the model. Their operation speed and accuracy are not the same. Based on the five-fold cross-validation results on the training dataset, LightGBM achieved the best overall performance among the candidate models. The optimized LightGBM model was subsequently evaluated using the independent testing dataset, achieving an accuracy of 0.818, recall of 0.818, precision of 0.909, and F1-score of 0.837 (Table 5).

**TABLE 5:** Comparison of machine learning model effects.

Model	Time(s)	Accuracy	Recall	Precision	F1
Random forest	0.077	0.636	0.636	0.879	0.675
AdaBoost	0.077	0.636	0.636	0.879	0.675
GBDT	0.283	0.636	0.636	0.879	0.675
Extra trees	0.083	0.545	0.545	0.727	0.606
CatBoost	0.09	0.727	0.727	0.891	0.758
XGBoost	0.356	0.636	0.636	0.716	0.674
LightGBM	0.036	0.818	0.818	0.909	0.837

F1 is a harmonic average of accuracy and recall.

**FIGURE 7:**
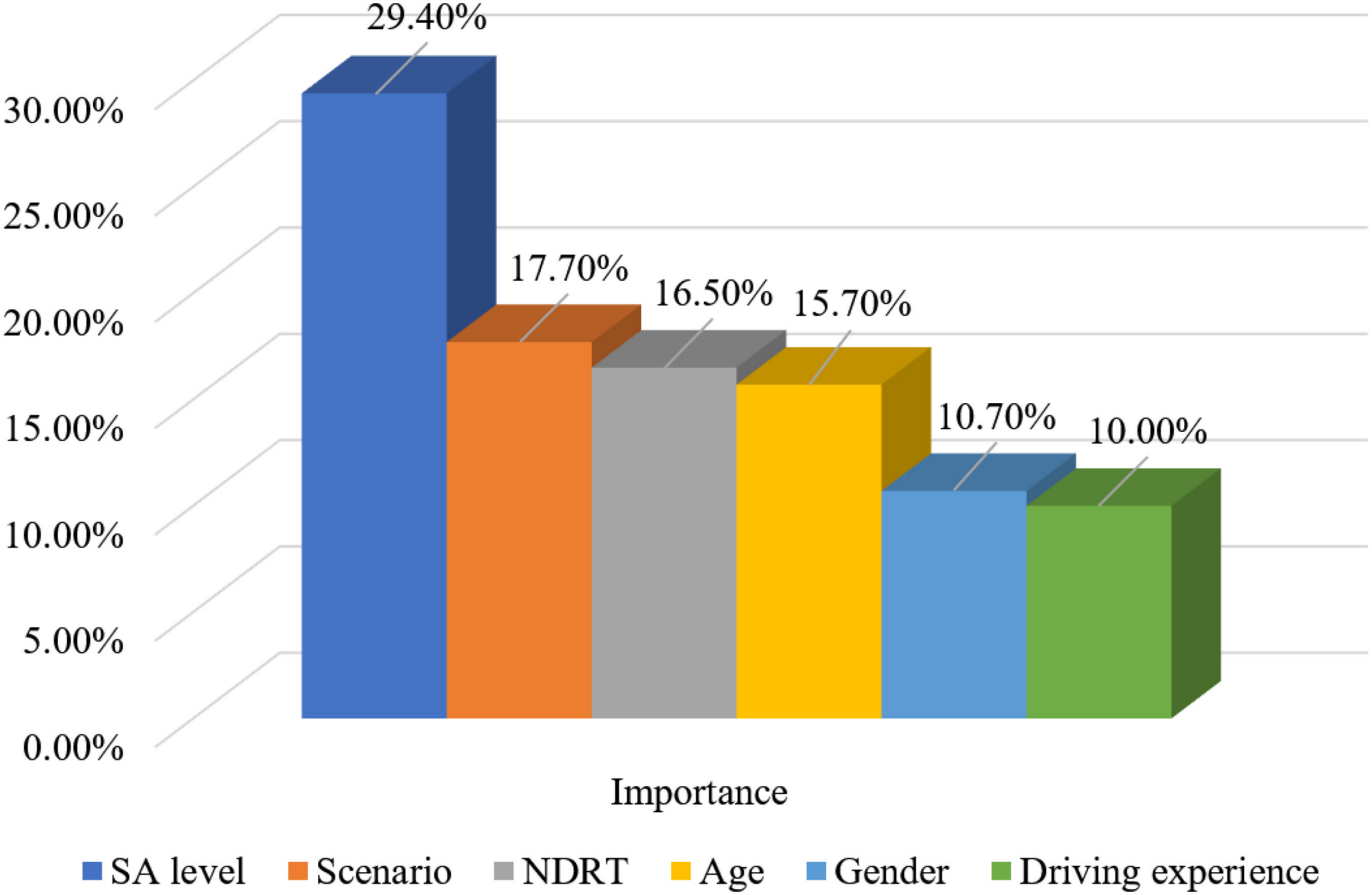
Contribution of factors influencing takeover reaction time.

LightGBM classification model was selected to predict reaction time and quantify the contribution of different factors, including the SA-related score (29.40%), takeover scenario (17.70%), NDRT (16.50%), age (15.70%), gender (10.70%), and driving experience (10.00%) (Figure 7). The results showed that among the investigated factors associated with takeover reaction time, the SA-related score exhibited the highest contribution. Higher SA-related scores were associated with shorter takeover reaction times (*p* < 0.05). Furthermore, different takeover scenarios and NDRTs were also associated with variations in reaction time. The reaction time under the control group’s mainline takeover condition was shorter than that under the ramp takeover condition. Similarly, the average reaction time under the work task condition was shorter than that under the recreational task condition. Regarding driver characteristics, age showed the greatest contribution and exhibited a V-shaped relationship with reaction time (p < 0.05), with young and older drivers demonstrating shorter reaction times than middle-aged drivers. Driving experience showed a similar pattern (*p* < 0.05). Gender contributed relatively less, and no significant difference was observed. Therefore, the SA-related score, takeover conditions, and driver age were identified as the major factors associated with takeover reaction time.

## 4 Discussion

By comparing the significance of various indicators across different takeover scenarios and NDRTs, this study found that NDRT type had no statistically significant effect on final takeover reaction time, which is consistent with the findings of Dogan et al. (2019). This phenomenon may be attributed to individual differences in cognitive absorption. Although participants were instructed to engage in NDRTs, the potential novelty effect of the driving simulator, where drivers remain subconsciously alert to the virtual road environment due to nervousness or curiosity, cannot be completely excluded. Therefore, different NDRTs were considered primarily as representing different levels of baseline cognitive demand. Regarding pre-takeover visual behavior, significant associations were observed between pupil area and fixation duration, as well as between fixation and saccade durations. These findings are consistent with cognitive load theory (Wickens, 2008). The variations in these eye-movement indicators across different NDRTs suggest that work-related and entertainment-based tasks may impose different effects on drivers’ visual information processing characteristics, supporting the findings of Lu et al. (2021). Furthermore, the LightGBM model revealed a nonlinear relationship between age and takeover-related characteristics, consistent with Zhang et al. (2023). By balancing the age distribution in our sample, middle-aged drivers exhibited relatively lower SA-related scores and longer reaction times. This finding differs from the conventional assumption of a monotonic decline in reaction speed with age. This phenomenon may be associated with differences in driving experience, cognitive strategies, and self-confidence among age groups, although further investigation is required to clarify the underlying mechanisms.

While the SA-related score showed the highest contribution to reaction time prediction, it should be noted that reaction time alone cannot fully represent overall takeover performance. As indicated by Marti et al. (2022), higher SA-related states and faster takeover responses do not necessarily guarantee safe and stable vehicle control. Takeover performance is a multidimensional concept involving not only response speed but also subsequent vehicle control quality, such as maximum deceleration, lane deviation, and operational stability. Therefore, the contribution of the proposed SA-related score should be interpreted as its association with takeover reaction time rather than a direct representation of its causal effect on overall takeover performance. Future research should integrate additional behavioral and vehicle-based indicators to further validate the relationship between drivers’ SA-related states and comprehensive takeover performance.

From a practical deployment perspective, while comprehensive eye-tracking provides high-resolution data for evaluating driver states, such complex instrumentation lacks ecological validity for mass-market vehicles. To realize dynamic takeover risk detection in the real world, data acquisition must become less intrusive and more accessible. Future safety systems could leverage lightweight, multimodal data streams. For instance, wearable devices (e.g., smartwatches) capturing electrocardiographic data, combined with historical driver profiles and real-time vehicle kinematics, could be used to infer SA. Ultimately, transitioning from theoretical predictive models to personalized, real-time takeover interventions represents a critical frontier for conditionally automated vehicle safety.

## 5 Conclusion

This study investigated the relationships between pre-takeover visual behaviors, drivers’ SA-related states, and takeover reaction time in a simulated Level 3 automated driving environment. By integrating pupil area, saccade duration, and fixation duration using the objective CRITIC weighting method, an eye-movement-based SA-related score was developed. Several machine-learning models were further compared, and the LightGBM model was used to evaluate the relative predictive contributions of the investigated factors to takeover reaction time. The main conclusions are summarized as follows:

(1) Different NDRTs influenced drivers’ pre-takeover visual processing characteristics. The statistical analysis showed that NDRT type significantly affected pupil area, saccade duration, and fixation duration, indicating differences in visual workload and information-processing demands under different secondary tasks. However, the difference in the resulting SA-related scores between the two NDRT conditions did not reach the conventional statistical significance threshold (*p* > 0.05). In addition, no significant difference was observed in takeover reaction time. These findings suggest that the effects of NDRTs were mainly reflected in individual pre-takeover eye-movement characteristics, whereas the differences in the integrated SA-related score and takeover reaction time should be interpreted cautiously.
(2) The proposed eye-movement-based SA-related score provides an objective representation of drivers’ pre-takeover visual processing states. By considering the relative importance and partially overlapping information among the eye-movement indicators, the CRITIC weighting method was used to integrate pupil area, saccade duration, and fixation duration into a comprehensive score. The results showed a statistically significant association between the SA-related score and takeover reaction time, indicating the potential of integrated eye-movement characteristics as a physiological proxy for estimating SA-related states during takeover preparation. Nevertheless, the proposed score should not be interpreted as a direct measurement of situational awareness as defined by Endsley.
(3) The SA-related score exhibited the highest predictive contribution to takeover reaction time among the investigated variables. The LightGBM results indicated that the eye-movement-based SA-related score contributed more substantially to reaction-time prediction than takeover scenario, NDRT type, and demographic characteristics. This finding highlights the importance of drivers’ pre-takeover visual information-processing characteristics in explaining variations in initial takeover response speed. However, this result represents a predictive association rather than evidence of a direct causal effect of SA-related states on takeover reaction time.
(4) The findings provide practical insights for adaptive driver monitoring and human–machine interaction systems in conditionally automated vehicles. By identifying variations in drivers’ visual processing states before a takeover request, the proposed approach may support personalized takeover assistance, such as adaptive warning timing and information presentation. It should be emphasized that the outcome examined in this study was takeover reaction time, defined as the interval between the warning trigger and pressing the takeover button, rather than the completion of control transfer, successful hazard avoidance, or overall takeover quality. Future studies should therefore incorporate additional physiological, behavioral, and vehicle-control indicators, such as maximum deceleration, lane deviation, steering stability, and time to stable manual control, to extend the assessment from initial takeover reaction time to comprehensive takeover performance.

## Data Availability

The raw data supporting the conclusions of this article will be made available by the authors, without undue reservation.
